# Profiling *Blautia* at high taxonomic resolution reveals correlations with cognitive dysfunction in Chinese children with Down syndrome

**DOI:** 10.3389/fcimb.2023.1109889

**Published:** 2023-02-10

**Authors:** Xueyu Hou, Na Wu, Shimeng Ren, Xinjuan Wang, Qing Mu, Yang Zhang, Shan Wang, Weidong Yu, Jingzhu Guo

**Affiliations:** ^1^ Department of Pediatrics, Peking University People’s Hospital, Beijing, China; ^2^ Department of Central Laboratory & Institute of Clinical Molecular Biology, Peking University People’s Hospital, Beijing, China; ^3^ Department of Pediatrics, The First Affiliated Hospital of Zhengzhou University, Zhengzhou, China; ^4^ Beijing Municipal Key Laboratory of Child Development and Nutriomics, Capital Institute of Pediatrics, Beijing, China

**Keywords:** *Blautia massiliensis*, *Blautia argi*, cognitive dysfunction, acetic acid, Down syndrome, *Blautia faecis*

## Abstract

**Introduction:**

Down syndrome (DS), the presence of a supernumerary chromosome 21, is associated with cognitive dysfunction caused by early neurodegenerative processes. Alterations in the gut microbiota were observed in Chinese children with DS, and the genus *Blautia* was associated with cognitive function in these children. Therefore, it is crucial to understand the detailed composition of this group at the species level and to explore the effect of specific species on cognitive function.

**Methods:**

In this study, *Blautia*-specific amplicon sequencing was conducted to identify the specific Blautia species in 15 children with DS and 15 matched healthy children.

**Results:**

The taxonomic analyses suggested that the *Blautia* taxa were clustered by disease status. The diversity of *Blautia* at the species level differed between DS patients and healthy controls, with the abundances of *Blautia* massiliensis and Blautia argi decreasing in DS children, while *Blautia faecis* was increased. Acetic acid, one of the metabolites of *Blautia*, was significantly reduced in the DS group. Of particular interest, Kyoto Encyclopaedia of Genes and Genomes analysis revealed decreased modules related to starch and sucrose metabolism and glycolysis. In addition, *B. argi* was positively related to DS cognitive scores, and *B. faecis* was negatively related to cognitive function, implying its role on the DS cognitive impairments.

**Discussion:**

Our study has important implications for understanding the important effects of specific species of Blautia on cognitive function and thus possibly provides a new strategy for future studies of cognitive improvement in individuals with DS.

## 1 Introduction

Trisomy 21, the presence of a supernumerary chromosome 21, results in a collection of clinical features commonly known as Down syndrome (DS) (Antonarakis et al., 2020). According to the Global Burden of Disease Report, DS accounted for approximately 2930 live births in China in 2019 (The global burden of disease (GBD) 2019 data). Children with DS commonly present cognitive dysfunction (Grieco et al., 2015) and are more likely to develop certain health conditions, including intellectual disability and early-​onset Alzheimer’s disease (AD). The cognitive profile of children with DS present with a distinct collection of symptoms, including cognitive dysfunction, bowel dysfunction and obesity (Squassante et al., 2015). Cognitive dysfunction greatly impairs patients’ quality of life and places heavy economic and mental pressure on their families and society.

The combined effects of genetic and environmental factors are thought to contribute to cognitive dysfunction. Recently, dysbiosis in gut microbiome composition has been suggested to be associated with neurodegenerative diseases, such as Alzheimer’s disease (AD) and Parkinson’s disease (PD). PD patients display distinct metabolic profiles in gut microbiota compared with those of healthy individuals, with alterations in metabolites associated with the gut microbiota, including reduced concentrations of short-chain fatty acids (SCFAs) (Unger et al., 2016; Ahmed et al., 2019). Alterations in gut microbiota composition have also been identified in individuals with AD, including a decreased abundance of Firmicutes, one of the core phyla of human gut microbiota (Biddle et al., 2013). As a genus within the Lachnospiraceae family in the Firmicutes phylum, *Blautia* has been of particular interest because of its contribution to metabolic diseases and for its antibacterial activity against specific microorganisms (Kalyana Chakravarthy et al., 2018). The *Blautia* genus has been found to have 25 subspecies (NCBI; Liu et al., 2021), which exhibit many differences in metabolic products and may play different roles in the disease process of neurodegenerative cognitive dysfunction (Fung et al., 2017). In our previous study, we observed the gut microbiota from DS patients showed different pattern compared with healthy controls. Besides, we identified that the *Blautia* genus was present at a lower relative abundance in the feces of children (Ren et al., 2022). As SCFA-producing bacteria, *Blautia* plays an important role in maintaining the environmental balance in the intestine and preventing inflammation. The microbiota-derived metabolite SCFAs were proven to have a protective role in cognitive functions Erny et al., 2021, and long-term acetic acid deficiency was suggested as a risk factor for cognitive decline (Zheng et al., 2021a), implying the potential of *Blautia* in cognitive function in individuals with DS (Chen et al., 2020).

However, most of these reports did not conduct in-depth studies at the species or even strain levels, and little is known about the detailed composition of this group in the gut microbiota of DS and healthy children. To avoid drawing general conclusions at the genus level, in this study, we used *Blautia* group-specific amplicon sequencing to investigate the composition of *Blautia* species in feces from the DS and healthy groups. Furthermore, a detailed correlation analysis was performed to provide important clinical clues based on the relative abundance of *Blautia* at the species level, levels of SCFAs and cognitive function in DS and healthy children.

## 2 Materials and methods

### 2.1 Ethical approval

The research scheme was approved by the Ethics Review Committee of Peking University People’s Hospital of China (Approval No:2019PHB110-01). All participants and their parents received oral and written information about the study and written consent was obtained before recruitment. All experiments were conducted in accordance with approved guidelines and regulations (Ren et al., 2022).

### 2.2 Participant recruitment

As described in our previous study (Ren et al., 2022), 15 children with DS were randomly recruited from integrated boarding schools in Beijing and its surrounding areas, and 15 age- and diet structure-matched children were recruited as the healthy group. Fifteen pairs of samples were collected for amplicon sequencing analysis. There were no differences in age and gender between DS group and healthy group. All the study participants had the same diet offered by school, which greatly reduces the impacts of diet, lifestyle and geography on gut microbiota profile. Besides, we excluded children who had received antibiotics within 3 months. Each participant’s height and weight were measured, and body mass index (BMI) was calculated by dividing weight by the square of height. The DS group had a lower average height (*P* = 0.037), a higher BMI (*P* = 0.009) than the healthy group. There was no difference in the weight between the two groups. Due to the limitation of the Chinese version of the Wechsler Intelligence Scale for Children (WISC-IV), only eleven pairs of participants over the age of 6 were subjected to the cognition test.

### 2.3 Assessments of cognitive function

To assess the participants’ cognitive function, we calculated the Full Scale IQ (FSIQ) test and measured the other four factor indices: the perceptual reasoning index (PRI), which was used to examine nonverbal fluid reasoning skills; the verbal comprehension index (VCI), which was used to measure verbal acquired knowledge and verbal reasoning; the working memory index (WMI), which was used to measure a person’s ability to manipulate linguistic information in short-term instant memory and then formulate responses; and the processing speed index (PSI), which was used to evaluate children’s ability to efficiently scan and understand visual information and use information to complete the work. Cognitive tests were conducted by two well-trained pediatricians in a hospital conference room with no interference. As described in our previous study (Ren et al., 2022), the scores of FSIQ, PRI, VCI, WMI and PSI were all decreased in the DS group (all *P* < 0.001). The correlation coefficient of this type is approximately 0.857.

### 2.4 Fecal sample collection and DNA extraction

Fecal bacterial DNA storage tubes (Longsee Biological Company, Guangzhou, China) were used to collect fecal samples. Before processing, samples are transferred and stored at − 80°C. The average moving time between sample collection and storage is 19 minutes at -80°C, and the maximum is 23 minutes. Total fecal DNA was extracted using a QIAamp DNA Stool Mini Kit (Qiagen, Hilden, Germany). The concentration of genomic DNA in each fecal sample was quantified using a NanoDrop 2000 spectrophotometer (Thermo Fisher Scientific, MA, USA). DNA integrity and sizes were assessed by 1% agarose gel electrophoresis. The DNA was resuspended in H_2_O and stored at −80°C prior to use.

The concentration detection results of the collected and extracted genomic DNA were all in line with the standards (**Table S1**). The purity, fragment size, and concentration of the amplified products of all samples met the sequencing requirements (**Figure S1**).

### 2.5 Quantitative PCR

Quantitative PCR (qPCR) amplifications were performed in a 20-μL reaction solution containing SYBR^®^ Green Real-time PCR Master Mix (Toyobo, Code No. QPK-201, Osaka, Japan). The specific primers for *Blautia* sp. covers all species of *Blautia* (**Figure S2**). The cycling conditions were as follows: 95°C for 1 min and then 33 cycles of 95°C for 15 s, 56°C for 15 s, and 72°C for 45 s.

The relative abundance of *Blautia* was evaluated using the following formulae. First, the relative value (RV) of *Blautia* was determined by the 2^−ΔΔct^ method with normalization to the total bacterial level. Then, the relative abundance (RA) of *Blautia* was scaled to a reasonable range with the following equation: RA = log_2_ (RV × 10000 + 1).

### 2.6 *Blautia*-specific amplicon sequencing

DNA extraction was performed for *Blautia*-specific PCR amplicon sequencing. Standard protocols were used to generate amplicons using a genus-specific primer from *Blautia*. Briefly, the hypervariable region of the 16S rRNA gene was amplified using a primer pair (477F: 5′-CGGTACCTGACTAAGAAGC-3′ and 719R: 5′-GTTCCTCCTAATATCTACGC-3′) with barcodes (Luu et al., 2017). All PCRs were performed with Phusion^®^ High-Fidelity PCR Master Mix (New England Biolabs). After the purification of a *Blautia*-specific PCR amplicon, the libraries were generated with an Ion Plus Fragment Library kit, and paired-end sequencing (2 × 125 bp) was performed with an IonS5™XL platform (Thermo Fisher) from Novogene (Beijing, China).

### 2.7 Sequence analysis

These reads were analyzed with QIIME ver. 2.2020.6 (Quantitative Insights into Microbial Ecology) software package. Single-end FASTQ reads were imported into QIIME using a q2 manifest file import method and quality filtered using the q2-dada2 denoising method. The frequencies of amplicon sequence variants (ASVs) were identified at the 100% similarity level. The representative sequences of ASVs were aligned with the Silva database. The taxonomic composition of each sample at the species level was calculated.

### 2.8 GC−MS analysis

Gas chromatography−mass spectrometry (GC−MS) analysis was conducted using a SHIMADZU GC2030-QP2020 NX GC−MS system with an HP-FFAP capillary column. A 1-μL aliquot of the analyte was injected in split mode (5:1). Helium was used as the carrier gas, the front inlet purge flow was 3 mL min^−1^, and the gas flow rate through the column was 1 mL min^−1^. The initial temperature was kept at 80°C for 1 min, then raised to 200°C at a rate of 10°C min^−1^ for 5 min, and then kept for 1 min at 240°C at a rate of 40°C min^−1^. The injection, transfer line, quad, and ion source temperatures were 240°C, 240°C, 200°C, and 150°C, respectively. The energy was −70 eV in electron impact mode. The mass spectrometry data were acquired in Scan mode with a m/z range of 33–150 after a solvent delay of 3.5 min.

### 2.9 Statistical analysis

The core metric phylogenetic method in the q2 diversity plug-in of QIME was used for diversity analysis. To estimate α-diversity (within sample), different metrics were calculated: Shannon (a quantitative measure of the number of ASVs present in a given sample and their relative abundance or community richness) and Simpson metrics. Alpha diversity indices were compared using the Mann–Whitney test. Principal coordinate analysis (PCoA) and Principal component analysis (PCA) based on the weighted UniFrac measures was used to visualize beta diversity relationships.

The microbial comparisons between the DS and healthy groups were performed using the Mann–Whitney test. Associations between clinical indices and *Blautia* were evaluated by the MaAsLin analysis (Stewart et al., 2018). Statistical analysis of the clinical data was performed using SPSS (Statistical Package for Social Sciences) 22.0 software (SPSS Inc., Chicago, IL, USA). *P* < 0.05 was considered statistically significant.

## 3 Results

### 3.1 *Blautia* species spectrum in DS and healthy children

A total of 2,400,845 high-quality combined sequence reads were obtained after data processing (average, 80,028.2 reads). All sequences were clustered into 150 representative ASVs, and 10 of the 150 ASVs were identified as *Blautia* taxa. The representative sequence of each ASV was assigned to the BLASTN dataset to estimate the diversity of *Blautia* species in the DS and healthy groups. Six published *Blautia* species with the best hits are shown in **Table 1**, namely, *B. massiliensis*, *B. wexlerae*, *B. faecis*, *B. stercoris*, *B. obeum*, and *B. argi*. Of these, ASV1, assigned to *B. massiliensis*, was the dominant species, representing 49.75% of *Blautia* sequences. In addition, the proportions of *B. wexlerae*, *B. faecis*, *B. stercoris*, *B. obeum*, and *B. argi* (ASV34 and ASV53) in the *Blautia* sequences were 41.85%, 2.98%, 2.54%, 2.10%, and 0.62%, respectively (ASV34 = 0.54%, ASV53 = 0.08%).

**Table 1:** Genomic DNA cluster numbering and taxonomic annotation.

#ASV ID	Taxonomy	DS mean	Healthy mean	DS/Healthy	Percentage
ASV1	*Blautia massiliensis*	0.0794	0.1822	0.436	49.7512%
ASV2	*Blautia wexlerae*	0.1035	0.1166	0.887	41.8526%
ASV17	*Blautia faecis*	0.0099	0.0057	1.73	2.9799%
ASV21	*Blautia stercoris*	0.0057	0.0077	0.743	2.5356%
ASV23	*Blautia obeum*	0.0057	0.0053	1.09	2.0959%
ASV34	*Blautia argi*	0.0008	0.0020	0.405	0.5421%
ASV49	*Blautia*	0.0005	0.0003	1.73	0.1599%
ASV53	*Blautia argi*	0	0.0004	0	0.0797%
ASV89	*Blautia*	0	1.17E-05	0	0.0022%
ASV143	*Blautia*	4.70E-06	0	NA	0.0009%

These findings revealed the detailed phylogenetic inventory of the *Blautia* bacterial group in the human gut microbiota and showed that *Blautia* is prevalent in fecal samples from DS and healthy children.

### 3.2 Differences in *Blautia* diversity between DS and healthy children

**Figure 1:**
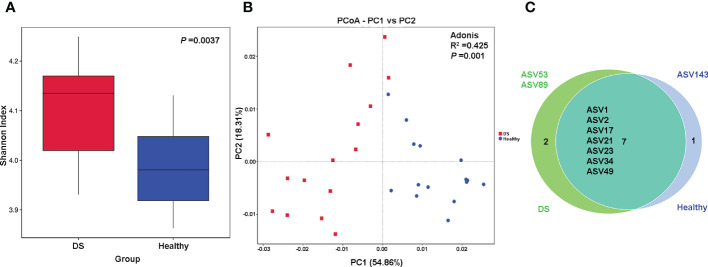
Differences in *Blautia* genus diversity between DS and healthy children. **(A)** Alpha diversity based on the Shannon index at the species level; **(B)** The principal coordinate analysis (PCoA) on the genus level; **(C)** Venn diagram illustrating the overlap of the ASVs identified in the fecal microbiota between the two groups.

Microbial alpha diversity was assessed by means of the Shannon and Simpson indices. The Shannon index was significantly increased in the DS group (**Figure 1A**) compared with the healthy group (4.104 vs. 3.984, *P* = 0.004), and there was no significant change of Simpson diversity index between the two groups (0.908 vs. 0.904, *P* = 0.1192) (**Figure S3A**), suggesting greater *Blautia* commensal diversity in the DS group. To further assess the alteration in the *Blautia* composition in the DS group, we used PCA to examine the observed discrete clustering of the intestinal microbiome in the DS and healthy groups. The results showed that the characteristics of the *Blautia* genus in DS patients were significantly different from those in healthy individuals (**Figure S3B**). Significant differences were also found by PCoA analysis, indicating that the *Blautia* genus structure differed significantly between the DS and healthy groups (**Figure 1B**). In addition, 7 of the 10 total ASVs were shared among all the samples. As shown in the Venn diagram, ASV143 was exclusively present in the DS group, while ASV53 (*Blautia argi*) and ASV89 were exclusively present in the healthy group (**Figure 1C**).

### 3.3 Differences in the relative abundance of *Blautia* species between DS and healthy children

**Figure 2:**
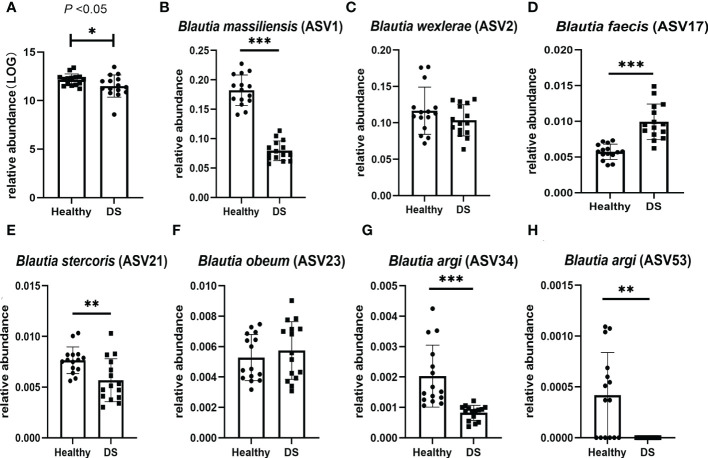
Differences in the relative abundance of *Blautia* species between DS and healthy children. **(A)** Relative abundance of *Blautia* between the two groups, *P<0.05 **(B**, **E**, **G**, **H)** Three species of *Blautia* were significantly decreased in children with DS, ***P < 0.0001, **P < 0.01; **(C**, **F)** Two species of *Blautia* were not significantly different between the two groups.

As a comparison of the group differences at the *Blautia* genus level, the quantitative PCR results showed that the relative abundance (RA) of *Blautia* was significantly decreased in the intestinal tract of children with DS (*P* < 0.05, **Figure 2A**). This result was consistent with our previous findings (Ren et al., 2022), which showed a significantly lower level of *Blautia* in children with DS. Taken together, these results indicate that the *Blautia* genus is disturbed in individuals with DS.

Next, we explored the RA of *Blautia* at the species level in DS and healthy children through *Blautia*-specific amplicon sequencing. The abundances of *B. wexlerae* and *B. obeum* were not significantly different between the two groups (**Figures 2C, F**).

Three species of *Blautia* were significantly decreased in children with DS: *B. massiliensis* (ASV1, *P* < 0.0001, **Figure 2B**), *B. stercoris* (ASV21, *P* = 0.0054, **Figure 2E**), and *B. argi* (ASV34, *P* = 0.0001, **Figure 2G**; ASV53, *P* = 0.0039, **Figure 2H**). Notably, *B. argi* (ASV53, *P* = 0.0039, **Figure 2H**) was nearly absent in children with DS. While, *B. faecis* significantly increased in children with DS (ASV17, P < 0.0001). While, B. faecis significantly increased in children with DS (ASV17, P < 0.0001, **Figure 2D**).

### 3.4 Relationship between *Blautia* species and cognitive function

**Figure 3:**
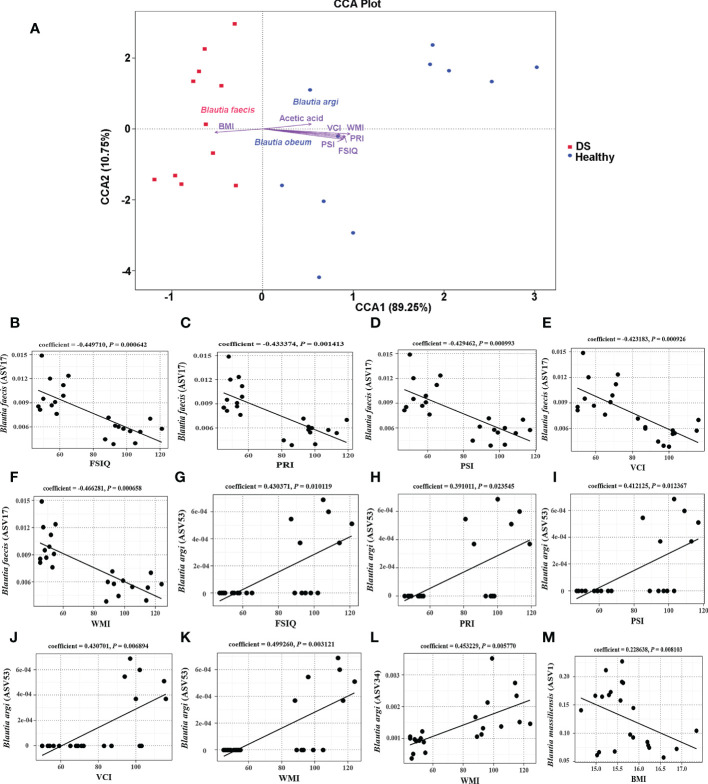
Correlations between *Blautia* species and cognitive function scores. **(A)** Key contributors to the *Blautia* variation were determined by CCA analysis. **(B–F)** The correlations between (*B*) *faecis* and all WISC-IV scores; **(G–K)** The correlations between (*B*) *argi* (ASV53) and all WISC-IV scores; **(L)** The correlation between (*B*) *argi* (ASV34) and the score of WMI; **(M)** The correlation between (*B*) *massiliensis* and BMI.

To explore the factors contributing to the *Blautia* variation in the gut microbiome of DS and healthy children, canonical correspondence analysis (CCA) was performed. Some clinical parameters were identified as important contributors for differentiating DS samples from healthy samples (**Figure 3A**, **Table S2**). Among them cognitive indices had significant effects on healthy subject clustering, indicating that these clinical parameters are highly associated with the *Blautia* community variation between DS and healthy children. WMI had the largest effect on the healthy sample variation (R^2^ = 0.798105, *P* = 0.005).

Furthermore, we examined correlations between the WISC-IV scores and the species level of *Blautia*. Significant negative correlations between *B. faecis* and all WISC-IV scores were observed by MaAsLin analysis (**Figures 3B–F**). *B. argi* (ASV53) had a significant positive association with all WISC-IV scores (**Figures 3G–K**), which is consistent with the findings of the CCA. While, *B. argi* (ASV34) was significant positively related to the score of WMI (**Figure 3L**). *B. argi* (ASV53) was indicated as an important contributor for differentiating healthy samples from DS samples. In addition, *B. massiliensis* had a significant positive association with BMI (**Figure 3M**). These results indicate that *B. argi* might be beneficial for cognitive function, while *B. faecis* might be a risk factor for cognitive function.

### 3.5 Relationship between *Blautia* species and acetic acid

**Figure 4:**
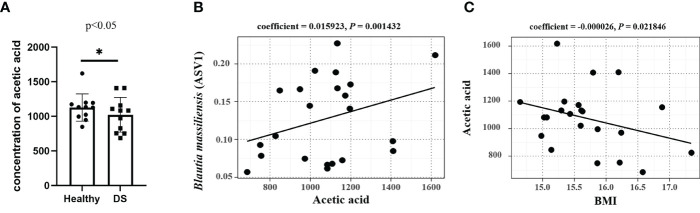
Correlations between (*B*) *massiliensis* and acetic acid. **(A)** The average level of acetic acid was significantly decreased in the feces of the DS group; **(B)** (*B*) *massiliensis* (ASV1) had a significant positive correlation with acetic acid contents; **(C)** The abundance of acetic acids had a significant negative correlation with BMI.

SCFAs, particularly acetic acid, propionate, and butyrate, are the end products of the intestinal microbial fermentation of dietary fibers and resistant starch. The levels of SCFAs were determined by GC−MS analysis (**Table S3**), and the average level of acetic acid was significantly lower in the feces of the DS group than in that of the healthy samples (*P* < 0.05, **Figure 4A**). Notably, *Blautia* has been reported to be an acetic acid-producing bacterium (Aoki et al., 2021), and *B. massiliensis* (ASV1) and acetic acid contents had a significant positive correlation (*P* = 0.0014, **Figure 4B**), indicating its acetic acid-producing property. The relative abundance of *Blautia* species showed a positive correlation with a high level of acetic acid, which is consistent with previous studies (Fung et al., 2017).

To further investigate the relationship between clinical indices and acetic acid, we used MaAsLin analysis to measure the correlation between WISC-IV scores, BMI and the level of acetic acid content. The findings indicated that acetic acid and *B. massiliensis* was negatively correlated with BMI (**Figures 3M** and **4C**), suggesting that acetic acid plays an important role in over-weight of DS children. However, our findings indicated no significant differences between the abundance of acetic acids and the WISC-IV scores.

The importance of the association between the proportion of *Blautia* species and a high level of acetic acid is therefore supported by the acetic acid-producing effect, implying a beneficial impact of *B. massiliensis* as an acetic acid producing species on cognitive (Wen et al., 2020) and metabolic function in Chinese children by the metabolites.

### 3.6 Predictive function analysis

**Figure 5:**
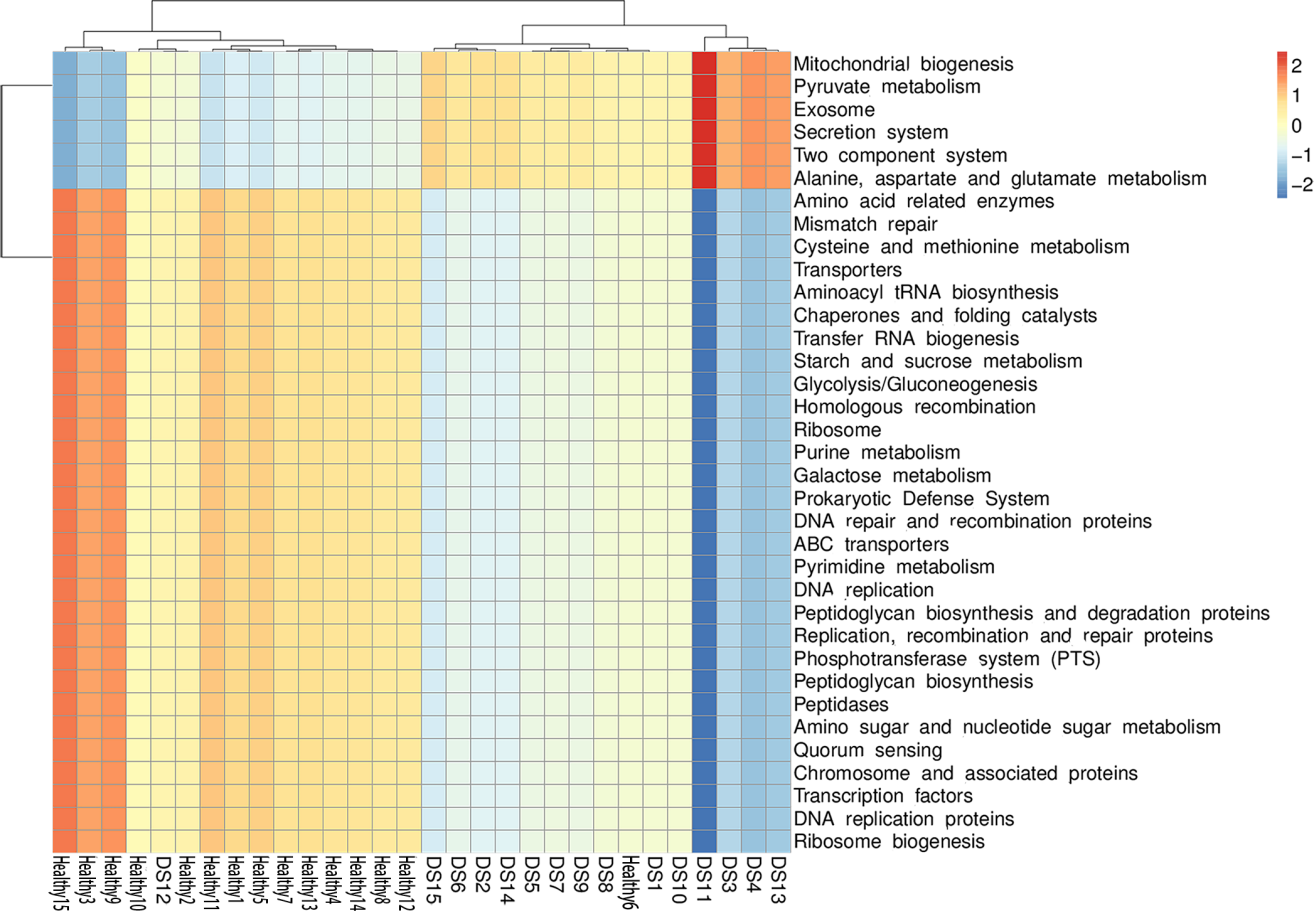
Functional predictions for the *Blautia* species of the DS and healthy control groups. Significantly enriched KEGG pathways at molecular level.

Tax4Fun, based on the closed-reference ASVs, was used to predict the functional categories of the KEGG Orthology (KO). The genomes of the *Blautia*-specific groups in DS children and healthy children were predicted, in conjunction with their physiological, cellular, and molecular functions (**Figure 5**). The genes of *Blautia* species in DS children were mainly enriched in molecular pathways, that is, pyruvate metabolism and glutamate metabolism. In contrast, the genes were deficient in the DS group in molecular pathways such as starch and sucrose and glycolysis metabolism. There were significant differences in the enrichment of the molecular pathways between the two groups of genes (**Figure S4**). Thus, functional genomics prediction suggested that *Blautia* may affect cognitive function by altering metabolites.

## 4 Discussion

Recent work has found that the gut microbiota play a key role in cognitive function in early neurodegenerative disease (Fung et al., 2017). In particular, the genus *Blautia*, one of the most abundant genera in the gut, has been identified to have an important relationship with DS and early-onset AD (Chen et al., 2020). In this study, the detailed composition of *Blautia* at species level in the gut microbiota of DS and healthy children was explored using *Blautia* group-specific amplicon sequencing. *B. massiliensis* were identified decreased in children with DS, and *B. argi* was nearly absent in children with DS, while *B. faecis* showed a higher level in DS children. In addition, we found that the level of *B. argi* was significantly correlated with cognitive WISC-IV scores. Our findings highlight the potential significance of *B. faecis* and *B. argi* in the development of cognitive function in children with DS and have implications for improving strategies in the gut-brain field. Molecular diversity at the species level with clinical cognitive function will help form the basis for future functional work of *Blautia* species on gut-brain neural mechanisms.

*Blautia* was proven to maintain the intestinal environmental balance by upregulating intestinal regulatory T cells and producing SCFAs (Chen et al., 2014). A cohort study (n = 108) reported that a lower proportion of *B. hansenii* could increase the risk of AD (Haran et al., 2019). A new study demonstrated that oral *Blautia stercoris MRx0006* can attenuate some of the behavioral deficits in an autism-relevant genetic mouse model (Sen et al., 2022). Additionally, a prospective study showed that the abundance of *Blautia* in PD patients was decreased, and the scores of neuropathological indicators were improved after fecal microbial transplantation with an increased abundance of *Blautia (* Kuai et al., 2021). Notably, we previously found that the genus *Blautia* was decreased in DS children and positively related to cognitive function (Ren et al., 2022), which suggests that different species of *Blautia* may exert beneficial effects on children with DS. To conduct a more in-depth study of *Blautia* at the species or even strain levels in the development of cognitive function in children with DS, we performed *Blautia*-specific amplicon sequencing and identified the profile and alteration of *Blautia* at the species level in Chinese children with DS. As expected, a shift of the *Blautia* group in individuals with DS was observed, as shown by the PCoA plot, with higher α-diversity and observed species in the Venn diagram. Abundances of several *Blautia* species were detected to differ in the DS and healthy groups. Among them, *B. massiliensis*, and *B. argi* were significantly decreased in DS samples. In addition, *B. massiliensis*, the dominant genus in healthy children samples, showed remarkably positive correlations with BMI. Another absent *Blautia* species in DS samples was *B. argi*, which was found to have a significant explanatory effect on the variation across healthy samples and showed a significant positive link with cognitive function. *B. argi* is a new species of *Blautia* isolated from dog feces (Paek et al., 2019). However, *B. argi* was present extremely low in healthy fecal samples and was at trace levels or even absent from the DS group. Further experiments are needed to verify its effect on cognitive function. Of particular interest, we revealed higher level of *B. faecis* in DS gut microbiota, which was also inversely correlated with WISC-IV scores, suggesting its negative role on host cognitive function. Furthermore, we found that acetic acid, a metabolite of *Blautia (* Flint et al., 2008), was decreased in the DS group and negatively correlated with BMI. Acetic acid, one of the major SCFAs, is metabolized by gut bacteria from dietary fiber fermentation and exerts a variety of physiological functions (Nicholson et al., 2012). It has been widely observed that acetic acid can promote metabolic syndrome *via* the gut–microbiota–brain–β cell axis (Perry et al., 2016). Moreover, acetic acid may enhance the efficiency of the synaptic vesicle cycle and thereby protect against cognitive impairment in mice with type 1 diabetes (Zheng et al., 2021b). Here, we observed that the relative abundance of *B. massiliensis* was positively associated with the level of acetic acid, indicating its acetic acid-producing ability. These findings suggest a probiotic role for the *Blautia* species *B. massiliensis* in the development with over-weight issue in children with DS through its metabolite product, especially acetic acid. Overall, this study provides potential insight for the promising application of *B. massiliensis* and acetic acid as preservatives of DS children with obesity issue in the future.

To reveal the genomic features of the alerted *Blautia* group with DS, we performed Tax4Fun analysis and predicted the genes of *Blautia* species in children with DS compared with healthy children. The pathways of pyruvate metabolism and glutamate metabolism were mainly enriched in DS intestinal samples. Pyruvate accumulation was reported to contribute to acceleration-induced impairment of cognitive abilities (Mo et al., 2021). Importantly, indole-3-pyruvic acid was identified as a signature for the discrimination and prediction of AD (Wu et al., 2021). Severe cognitive deficit correlates with high levels of lactate and pyruvate in cerebrospinal fluid according to the senile dementia report (Pugliese et al., 2005). Pyruvate can be metabolized to glutamine in the brain (Gray et al., 2014). The synthesis of glutamine mainly occurs in astrocytes and is related to the immune inflammatory response of the nervous system (Storm-Mathisen et al., 1983). In addition, we also observed that the genes of *Blautia* species related to glycolysis were decreased in children with DS. The major function of aerobic glycolysis is to maintain high levels of glycolytic intermediates to support anabolic reactions in cells (Lunt and Vander Heiden, 2011). Recent studies revealed that changes in aerobic glycolysis prevail in the early phase of AD (An et al., 2018). Astrocytic glycolysis affects cognitive functions, and dysregulations of glycolysis cause synaptic plasticity and behavioral deficits in AD (Le Douce et al., 2020). The reduction in glycolysis led by the decreased abundance of *Blautia* species further provides potential insight for the possible application of *Blautia* species and metabolites in the future for delaying and ameliorating cognitive dysfunction in children with DS. Another interesting observation is the decreased starch and sucrose metabolism pathway in the DS group, which had the same trend as *B. massiliensis* in the DS group. *B. massiliensis* also present a significant negative correlation with BMI index. Comparative genomics studies also showed that the genes encoding *B. massiliensis* were mainly enriched in carbohydrate transport and metabolism functions compared with other species within the genus *Blautia (* Durand et al., 2017). The final products of glucose fermentation by *B. massiliensis* include acetic acid, succinic acid, and lactic acid (Liu et al., 2021). Taken together, the decrease in *B. massiliensis* may affect the cognitive function and weight of children with DS by reducing carbohydrate metabolism, especially acetic acid from glucose metabolism. These findings indicate that pyruvate and glutamate metabolism abnormalities due to *Blautia* species deletion may have a major impact on cognitive dysfunction in DS.

In summary, cognitive improvements made during a small window early in life are important for individuals with DS (Antonarakis et al., 2020), and the gut microbiota has potential in the prevention and treatment of early neurodegenerative disease (Heiss and Olofsson, 2019). Our study profiled *Blautia* at the species level and suggested that two *Blautia* species, *B. faecis* and *B. argi*, have a close relationship with cognitive function in children with DS. Uniquely, in the present study, the data indicate that the *Blautia* group in subjects with DS is correlated with cognitive function, providing a deeper understanding that modifying the proportion of *B. faecis* and *B. argi*, may help ameliorating cognitive dysfunction for children with DS. Additionally, we highlight the specific probiotic and *Blautia*-derived product acetic acid and *B. massiliensis*, with plausible effects on obesity for DS children. Modulating the profile of the acetate-producing *Blautia* taxa, could be a promising strategy in the search for alternatives for the improvement of cognitive function and metabolic disorders in children with DS.

Our research is limited (1) by the lack of macro genome sequencing data to identify important species’ metabolic pathways, (2) by the limited sample size, and further studies with more individuals needed to confirm our findings. Furthermore, our suggestion on the prevalence of specific species with different metabolites requires future research to reveal the metabolic pathways of these species in the DS gut microbiota. Long-term observation may be helpful to study the dynamic changes in the *Blautia* group in the intestinal microbiota of children with DS at the species level during the growth process.

## 5 Conclusion

In conclusion, our data extend our previous work and provide evidence for the molecular diversity of *Blautia* species in the fecal microbiota of DS patients compared with healthy subjects. Importantly, we identified three species, *B. massiliensis, B. argi* and *B. faecis*, that may play an important role in the development of cognitive function in children with DS.

## Data availability statement

The datasets presented in this study can be found in online repositories. The names of the repository/repositories and accession number(s) can be found below: https://www.ncbi.nlm.nih.gov/bioproject/PRJNA885084/, PRJNA885084.

## Ethics statement

The studies involving human participants were reviewed and approved by the Ethics Review Committee of Peking University People’s Hospital of China. Written informed consent to participate in this study was provided by the participants’ legal guardian/next of kin.

## Author contributions

JG, XH, and SR contributed to conception and design of the study. SR and XW collected samples. XH, QM, and YZ extracted the sample DNA. XH and NW performed the statistical analysis. XH and NW wrote the first draft of the manuscript. XH, NW, WY, SW, and JG wrote sections of the manuscript. All authors contributed to the article and approved the submitted version.

## References

[B1] Ahmed S. Busetti A. Fotiadou P. Vincy Jose N. Reid S. Georgieva M.. (2019). *In vitro* characterization of gut microbiota-derived bacterial strains with neuroprotective properties. Front. Cell Neurosci., 13:402. doi: 10.3389/fncel.2019.00402 31619962 PMC6763572

[B2] An Y. Varma V. R. Varma S. Casanova R. Dammer E. Pletnikova O.. (2018). Evidence for brain glucose dysregulation in alzheimer's disease. Alzheimers Dement. 14 (3), 318–329. doi: 10.1016/j.jalz.2017.09.011 29055815 PMC5866736

[B3] Antonarakis S. E. Skotko B. G. Rafii M. S. Strydom A. Pape S. E. Bianchi D. W.. (2020). Down syndrome. Nat. Rev. Dis. Primers. 6 (1), 9. doi: 10.1038/s41572-019-0143-7 32029743 PMC8428796

[B4] Aoki R. Onuki M. Hattori K. Ito M. Yamada T. Kamikado K.. (2021). Commensal microbe-derived acetate suppresses NAFLD/NASH development *via* hepatic FFAR2 signalling in mice. Microbiome. 9 (1), 188. doi: 10.1186/s40168-021-01125-7 34530928 PMC8447789

[B5] The global burden of disease (GBD) 2019 data. Available at: http://ghdx.healthdata.org/gbd-results-tool.

[B6] Biddle A. Stewart L. Blanchard J. Leschine S. (2013). Untangling the genetic basis of fibrolytic specialization by lachnospiraceae and ruminococcaceae in diverse gut communities. Diversity (Basel). 5 (3), 627–640. doi: 10.3390/d5030627

[B7] Chen Y. Fang L. Chen S. Zhou H. Fan Y. Lin L.. (2020). Gut microbiome alterations precede cerebral amyloidosis and microglial pathology in a mouse model of alzheimer's disease. BioMed. Res. Int. 2020, 8456596. doi: 10.1155/2020/8456596 32596386 PMC7273394

[B8] Chen L. Wang W. Zhou R. Ng S. C. Li J. Huang M.. (2014). Characteristics of fecal and mucosa-associated microbiota in Chinese patients with inflammatory bowel disease. Med. (Baltimore). 93 (8), e51. doi: 10.1097/MD.0000000000000051 PMC4602441 25121355

[B9] Durand G. A. Pham T. Ndongo S. Traore S. I. Dubourg G. Lagier J. C.. (2017). *Blautia massiliensis* sp. *nov.*, isolated from a fresh human fecal sample and emended description of the genus *Blautia* . Anaerobe. 43, 47–55. doi: 10.1016/j.anaerobe.2016.12.001 27923606

[B10] Erny D. Dokalis N. Mezö C. Castoldi A. Mossad O. Staszewski O.. (2021). Microbiota-derived acetate enables the metabolic fitness of the brain innate immune system during health and disease. Cell Metab. 33 (11), 2260–2276.e7. doi: 10.1016/j.cmet.2021.10.010 34731656

[B11] Flint H. J. Bayer E. A. Rincon M. T. Lamed R. White B. A. (2008). Polysaccharide utilization by gut bacteria: potential for new insights from genomic analysis. Nat. Rev. Microbiol. 6 (2), 121–131. doi: 10.1038/nrmicro1817 18180751

[B12] Fung T. C. Olson C. A. Hsiao E. Y. (2017). Interactions between the microbiota, immune and nervous systems in health and disease. Nat. Neurosci. 20 (2), 145–155. doi: 10.1038/nn.4476 28092661 PMC6960010

[B13] Gray L. R. Tompkins S. C. Taylor E. B. (2014). Regulation of pyruvate metabolism and human disease. Cell Mol. Life Sci. 71 (14), 2577–2604. doi: 10.1007/s00018-013-1539-2 24363178 PMC4059968

[B14] Grieco J. Pulsifer M. Seligsohn K. Skotko B. Schwartz A. (2015). Down syndrome: Cognitive and behavioral functioning across the lifespan. Am. J. Med. Genet. C Semin. Med. Genet. 169 (2), 135–149. doi: 10.1002/ajmg.c.31439 25989505

[B15] Haran J. P. Bhattarai S. K. Foley S. E. Dutta P. Ward D. V. Bucci V.. (2019). Alzheimer's disease microbiome is associated with dysregulation of the anti-inflammatory p-glycoprotein pathway. mBio. 10 (3), e00632–e00619. doi: 10.1128/mBio.00632-19 31064831 PMC6509190

[B16] Heiss C. N. Olofsson L. E. (2019). The role of the gut microbiota in development, function and disorders of the central nervous system and the enteric nervous system. J. Neuroendocrinol. 31 (5), e12684. doi: 10.1111/jne.12684 30614568

[B17] Kalyana Chakravarthy S. Jayasudha R. Sai Prashanthi G. Ali M. H. Sharma S. Tyagi M.. (2018). Dysbiosis in the gut bacterial microbiome of patients with uveitis, an inflammatory disease of the eye. Indian J. Microbiol. 58 (4), 457–469. doi: 10.1007/s12088-018-0746-9 30262956 PMC6141402

[B18] Kuai X. Y. Yao X. H. Xu L. J. Zhou Y. Q. Zhang L. P. Liu Y.. (2021). Evaluation of fecal microbiota transplantation in parkinson's disease patients with constipation. Microb. Cell Fact. 20 (1), 98. doi: 10.1186/s12934-021-01589-0 33985520 PMC8120701

[B19] Le Douce J. Maugard M. Veran J. Matos M. Jégo P. Vigneron P. A.. (2020). Impairment of glycolysis-derived l-serine production in astrocytes contributes to cognitive deficits in alzheimer's disease. Cell Metab. 31 (3), 503–517.e8. doi: 10.1016/j.cmet.2020.02.004 32130882

[B20] Liu X. Mao B. Gu J. Wu J. Cui S. Wang G.. (2021). *Blautia*-a new functional genus with potential probiotic properties? Gut Microbes 13 (1), 1–21. doi: 10.1080/19490976.2021.1875796 PMC7872077 33525961

[B21] Lunt S. Y. Vander Heiden M. G. (2011). Aerobic glycolysis: meeting the metabolic requirements of cell proliferation. Annu. Rev. Cell Dev. Biol. 27, 441–464. doi: 10.1146/annurev-cellbio-092910-154237 21985671

[B22] Luu T. H. Michel C. Bard J. M. Dravet F. Nazih H. Bobin-Dubigeon C. (2017). Intestinal proportion of *Blautia* sp. is associated with clinical stage and histoprognostic grade in patients with early-stage breast cancer. Nutr. Cancer. 69 (2), 267–275. doi: 10.1080/01635581.2017.1263750 28094541

[B23] Mo F. Zhang H. Tang Y. Qi R. Nie S. Shen H.. (2021). Pyruvate accumulation may contribute to acceleration-induced impairment of physical and cognitive abilities: an experimental study. Biosci. Rep. 41 (4), BSR20204284. doi: 10.1042/BSR20204284 33782696 PMC8047541

[B24] NCBI. Available at: https://www.ncbi.nlm.nih.gov/data-hub/taxonomy/tree/?taxon=572511.

[B25] Nicholson J. K. Holmes E. Kinross J. Burcelin R. Gibson G. Jia W.. (2012). Host-gut microbiota metabolic interactions. Science. 336 (6086), 1262–1267. doi: 10.1126/science.1223813 22674330

[B26] Paek J. Shin Y. Kook J. K. Chang Y. H. (2019). *Blautia argi* sp. *nov.*, a new anaerobic bacterium isolated from dog faeces. Int. J. Syst. Evol. Microbiol. 69 (1), 33–38. doi: 10.1099/ijsem.0.002981 30407903

[B27] Perry R. J. Peng L. Barry N. A. Cline G. W. Zhang D. Cardone R. L.. (2016). Acetic acid mediates a microbiome-brain-β-cell axis to promote metabolic syndrome. Nature. 534 (7606), 213–217. doi: 10.1038/nature18309 27279214 PMC4922538

[B28] Pugliese M. Carrasco J. L. Andrade C. Mas E. Mascort J. Mahy N. (2005). Severe cognitive impairment correlates with higher cerebrospinal fluid levels of lactate and pyruvate in a canine model of senile dementia. Prog. Neuropsychopharmacol. Biol. Psychiatry 29 (4), 603–610. doi: 10.1016/j.pnpbp.2005.01.017 15866364

[B29] Ren S. Wang X. Qin J. Mu Q. Ye S. Zhang Y.. (2022). Altered gut microbiota correlates with cognitive impairment in Chinese children with down's syndrome. Eur. Child Adolesc. Psychiatry 31 (1), 189–202. doi: 10.1007/s00787-021-01799-2 33999314 PMC8816804

[B30] Sen P. Sherwin E. Sandhu K. Bastiaanssen T. F. S. Moloney G. M. Golubeva A.. (2022). The live biotherapeutic *Blautia stercoris MRx0006* attenuates social deficits, repetitive behaviour, and anxiety-like behaviour in a mouse model relevant to autism. Brain Behav. Immun. 106, 115–126. doi: 10.1016/j.bbi.2022.08.007 35995237

[B31] Squassante L. Spiridigliozzi G. Visootsak J. Heller J. Khwaja O. (2015). Assessment of cognitive scales to examine memory, executive function and language in individuals with down syndrome: Implications of a 6-month observational study. Front. Behav. Neurosci. 9, 300. doi: 10.3389/fnbeh.2015.00300 26635554 PMC4650711

[B32] Stewart C. J. Ajami N. J. O'Brien J. L. Hutchinson D. S. Smith D. P. Wong M. C.. (2018). Temporal development of the gut microbiome in early childhood from the TEDDY study. Nature. 562 (7728), 583–588. doi: 10.1038/s41586-018-0617-x 30356187 PMC6415775

[B33] Storm-Mathisen J. Leknes A. K. Bore A. T. Vaaland J. L. Edminson P. Haug F. M.. (1983). First visualization of glutamate and GABA in neurones by immunocytochemistry. Nature. 301 (5900), 517–520. doi: 10.1038/301517a0 6130475

[B34] Unger M. M. Spiegel J. Dillmann K. U. Grundmann D. Philippeit H. Bürmann J.. (2016). Short chain fatty acids and gut microbiota differ between patients with parkinson's disease and age-matched controls. Parkinsonism Relat. Disord. 32, 66–72. doi: 10.1016/j.parkreldis.2016.08.019 27591074

[B35] Wen C. Xie T. Pan K. Deng Y. Zhao Z. Li N.. (2020). Acetate attenuates perioperative neurocognitive disorders in aged mice. Aging (Albany NY). 12 (4), 3862–3879. doi: 10.18632/aging.102856 32139660 PMC7066918

[B36] Wu L. Han Y. Zheng Z. Peng G. Liu P. Yue S.. (2021). Altered gut microbial metabolites in amnestic mild cognitive impairment and alzheimer's disease: Signals in host-microbe interplay. Nutrients. 13 (1), 228. doi: 10.3390/nu13010228 33466861 PMC7829997

[B37] Zheng H. Xu P. Jiang Q. Xu Q. Zheng Y. Yan J.. (2021a). Depletion of acetate-producing bacteria from the gut microbiota facilitates cognitive impairment through the gut-brain neural mechanism in diabetic mice. Microbiome. 9 (1), 145. doi: 10.1186/s40168-021-01088-9 34172092 PMC8235853

[B38] Zheng H. Xu P. Jiang Q. Xu Q. Zheng Y. Yan J.. (2021b). Depletion of acetic acid-producing bacteria from the gut microbiota facilitates cognitive impairment through the gut-brain neural mechanism in diabetic mice. Microbiome. 9 (1), 145. doi: 10.1186/s40168-021-01088-9 34172092 PMC8235853

