# Supplementary figures and images for: Profiling Blautia at high taxonomic resolution reveals correlations with cognitive dysfunction in Chinese children with Down syndrome
Source: Front Cell Infect Microbiol. 2023 Feb 10;13:1109889. doi: 10.3389/fcimb.2023.1109889 (PMC9950735; doi:10.3389/fcimb.2023.1109889)

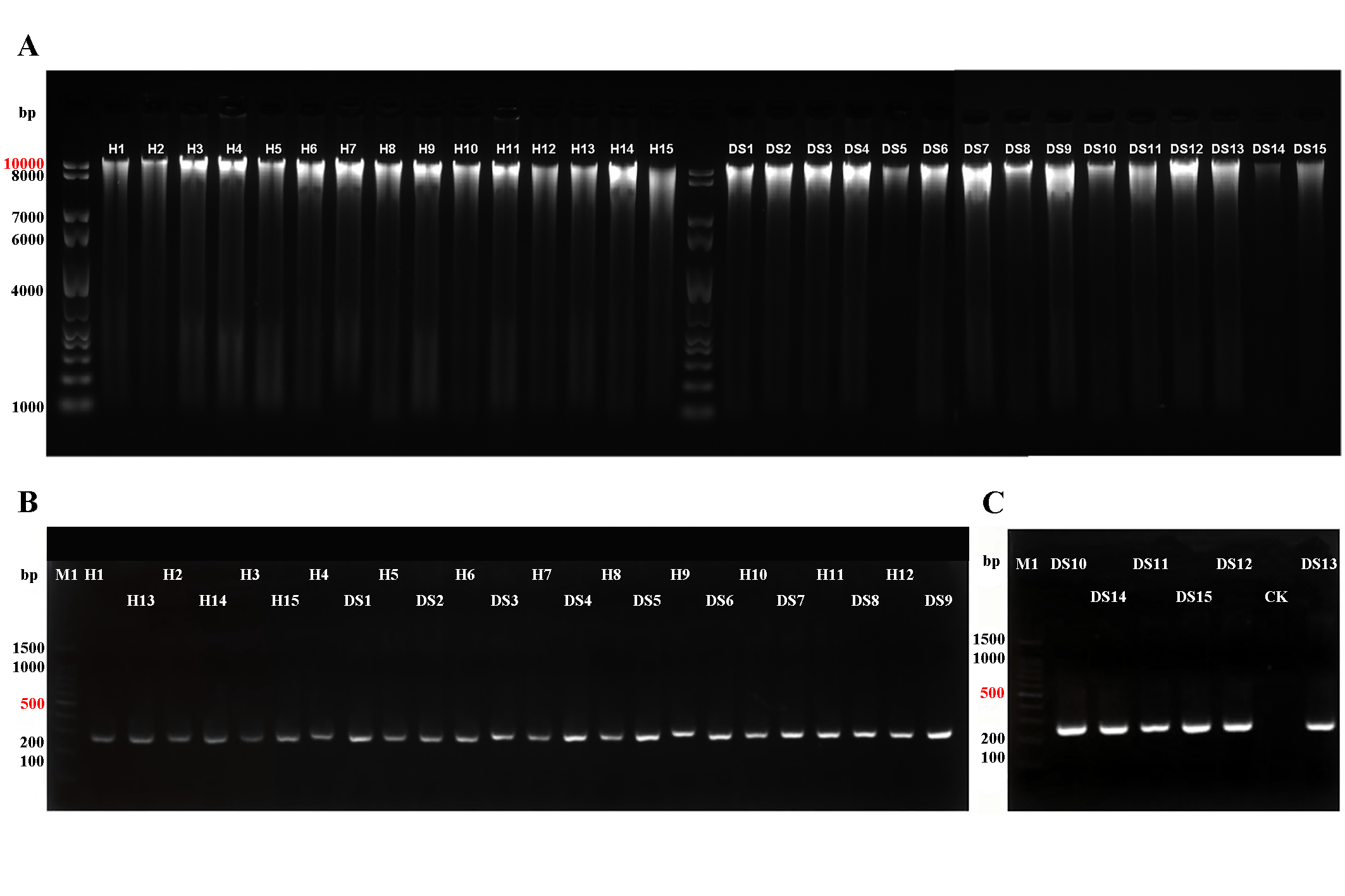

Supplement: Supplementary file 1 [file Image_1.tif]

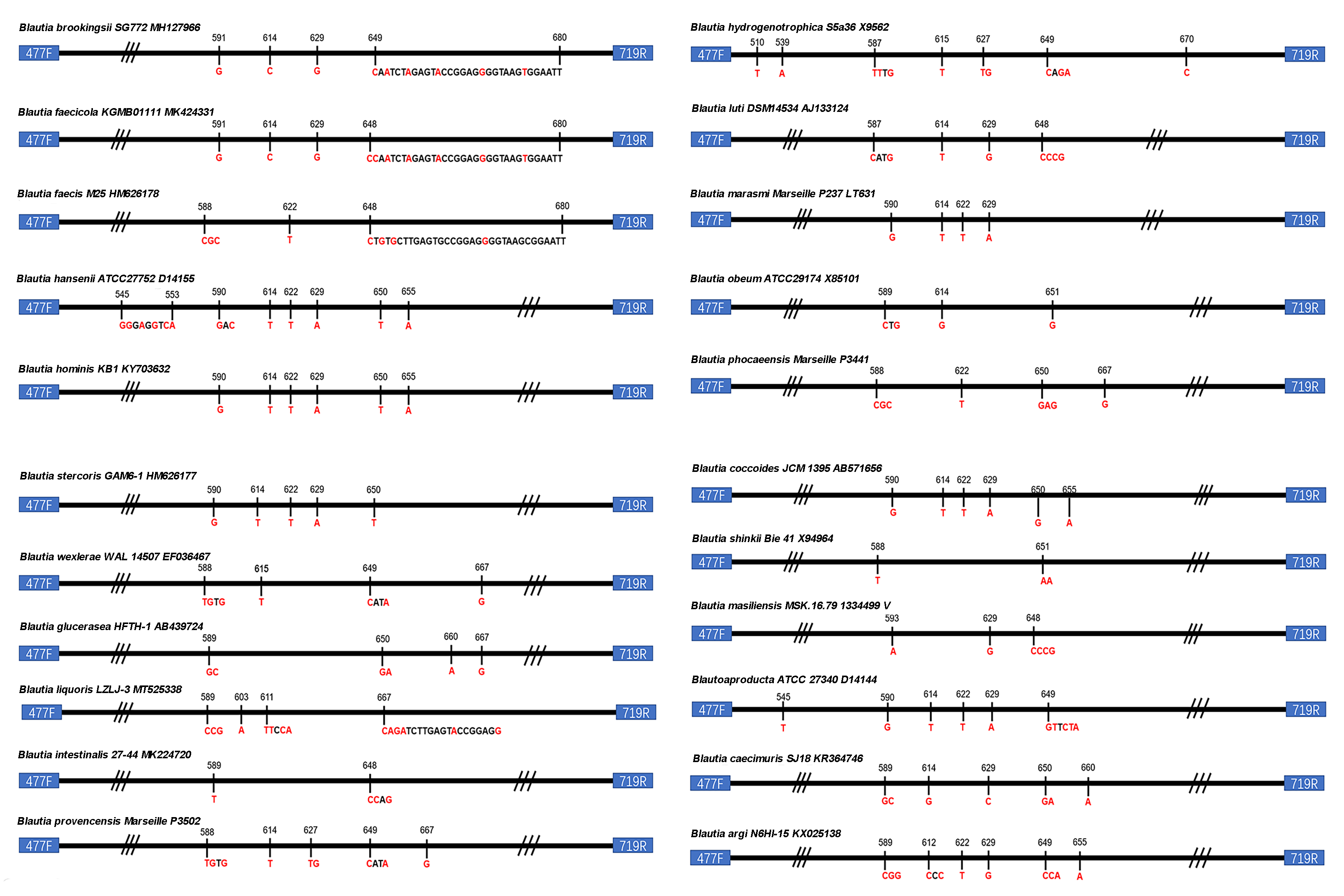

Supplement: Supplementary file 2 [file Image_2.tif]

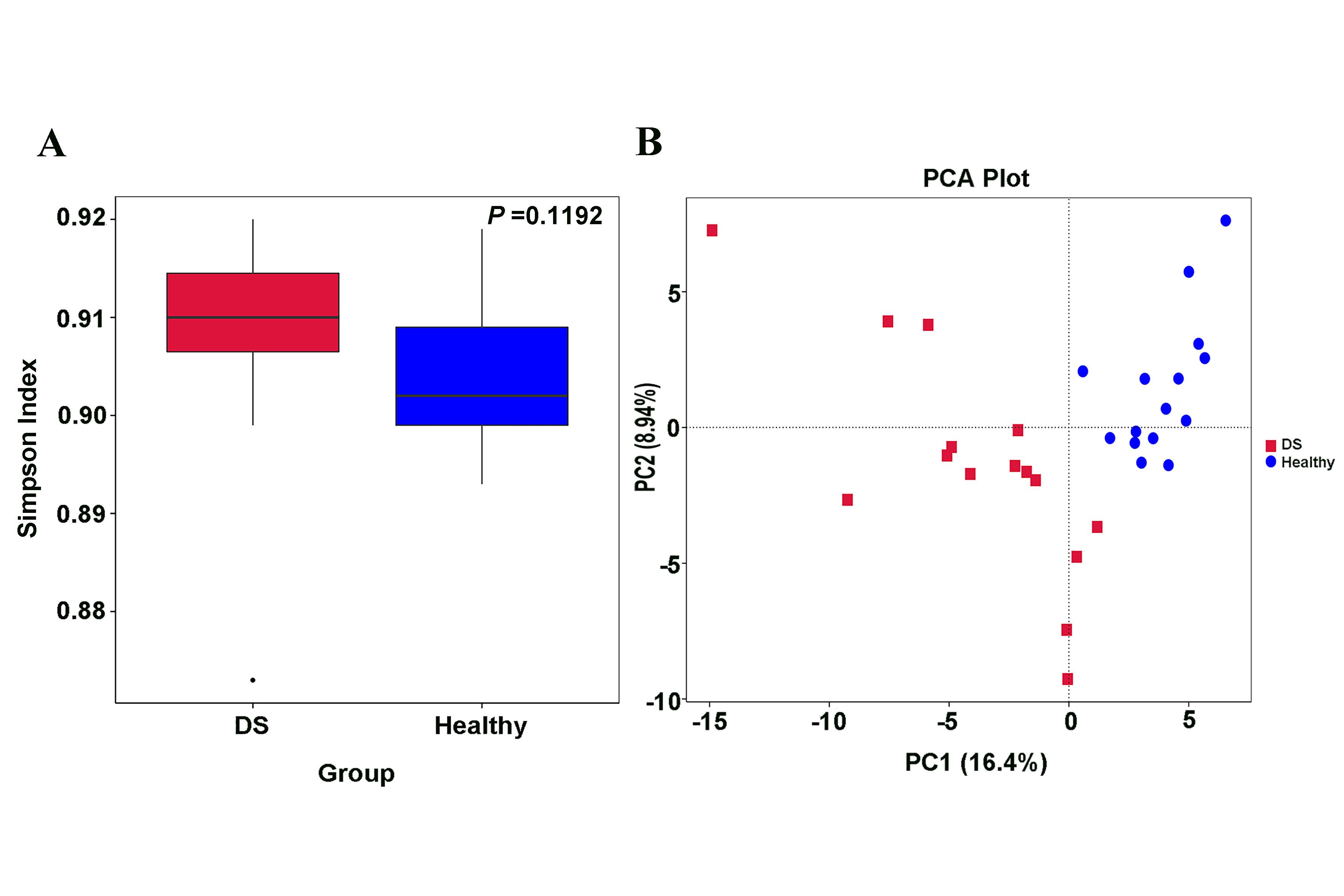

Supplement: Supplementary file 3 [file Image_3.tif]

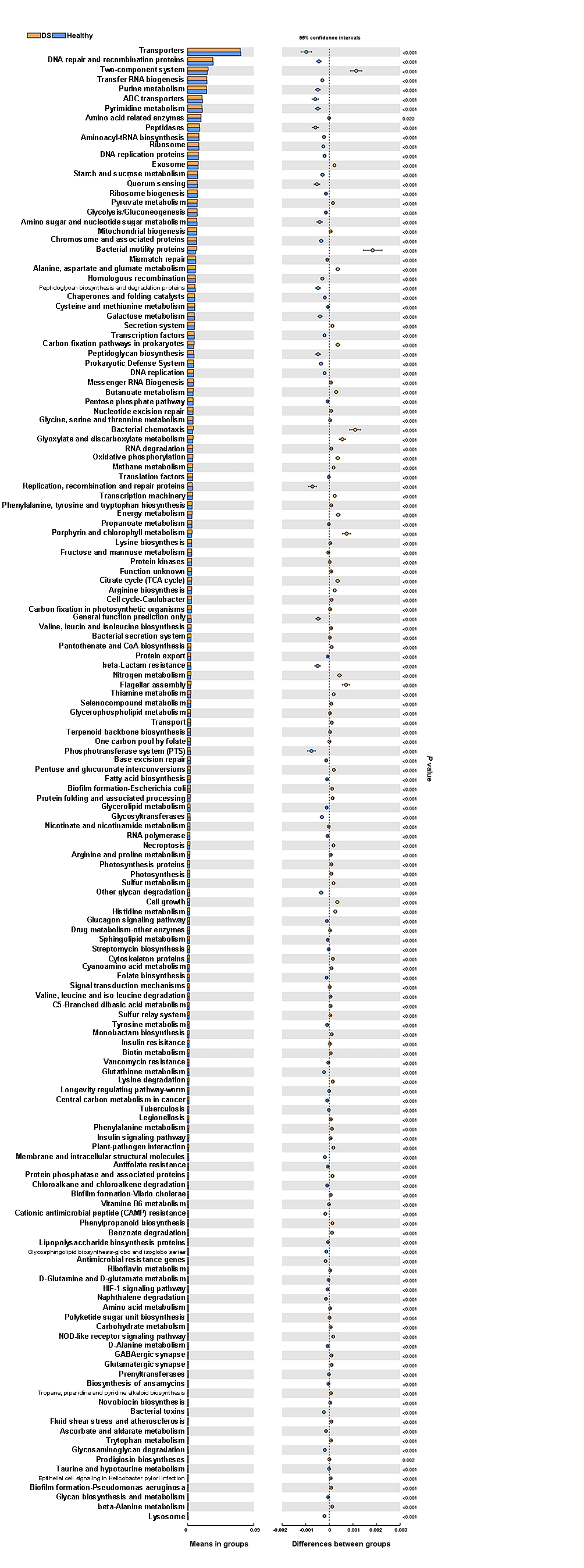

Supplement: Supplementary file 4 [file Image_4.tif]
