# Supplementary material for: Profiling Blautia at high taxonomic resolution reveals correlations with cognitive dysfunction in Chinese children with Down syndrome
Source: Front Cell Infect Microbiol. 2023 Feb 10;13:1109889. doi: 10.3389/fcimb.2023.1109889 (PMC9950735; doi:10.3389/fcimb.2023.1109889)
Supplement: Supplementary file 5 [file DataSheet_1.docx]

Supplementary Material

Profiling *Blautia* at high taxonomic resolution reveals the correlations with cognitive dysfunction in Chinese children with Down syndrome

Xueyu Hou^1†^, Na Wu^2†^, Shimeng Ren^3†^, Xinjuan Wang^2^, Qing Mu^2^, Yang Zhang^1^, Shan Wang^4^, Weidong Yu^2*^, Jingzhu Guo^1*^

**†These authors contributed equally to this work and share first authorship.**

*** Correspondence:**

Prof. Jingzhu Guo: Department of Pediatrics, Peking University People’s Hospital, No.11 Xizhimen South Street, Xicheng District, Beijing, China. Postal code: 100044,

E-mail: jingzhu.guo@bjmu.edu.cn

Prof. Weidong Yu: Department of Central Laboratory & Institute of Clinical Molecular Biology, Peking University People’s Hospital, No.11 Xizhimen South Street, Xicheng District, Beijing, China. Postal code: 100044,

E-mail: [weidongyu@bjmu.edu.cn](mailto:weidongyu@bjmu.edu.cn)

# Supplementary Figures and Tables

## Supplementary Figures

**Supplementary Figure 1.** The purity, fragment size, and concentration of the amplified products of all samples met the sequencing requirements.

**Supplementary Figure 2.** The specific primers for *Blautia* *sp*. covers all species of *Blautia*.

**Supplementary Figure 3.** The diversity of *Blautia* genus between two groups

**Supplementary Figure 4.** There were significant differences in the enrichment of the molecular pathways between the two groups of genes.

## Supplementary Tables

Table S1 Concentrations of collected and extracted genomic DNA

| Sample | DNA concentration  (ng/µL) | | A260/A280 | | Sample | DNA concentration (ng/µL) | | A260/A280 |
| --- | --- | --- | --- | --- | --- | --- | --- | --- |
| A1 | | 94.597 | | 1.864 | B1 | 129.604 | 1.868 | |
| A2 | | 146.137 | | 1.843 | B2 | 192.743 | 1.864 | |
| A3 | | 168.452 | | 1.846 | B3 | 153.73 | 1.848 | |
| A4 | | 218.834 | | 1.847 | B4 | 148.763 | 1.862 | |
| A5 | | 163.438 | | 1.844 | B5 | 52.307 | 1.774 | |
| A6 | | 194.44 | | 1.846 | B6 | 146.969 | 1.848 | |
| A7 | | 190.361 | | 1.839 | B7 | 233.945 | 1.849 | |
| A8 | | 127.56 | | 1.848 | B8 | 120.226 | 1.805 | |
| A9 | | 198.213 | | 1.852 | B9 | 219.829 | 1.851 | |
| A10 | | 83.335 | | 1.758 | B10 | 86.862 | 1.822 | |
| A11 | | 209.785 | | 1.862 | B11 | 209.268 | 1.854 | |
| A12 | | 108.755 | | 1.857 | B12 | 183.101 | 1.858 | |
| A13 | | 141.114 | | 1.846 | B13 | 174.127 | 1.843 | |
| A14 | | 255.941 | | 1.841 | B14 | 17.9 | 1.84 | |
| A15 | | 137.743 | | 1.857 | B15 | 53 | 1.87 | |

Table S2 CCA environmental fit

| Environmental factor | R2 | P value |
| --- | --- | --- |
| Age | 0.011319 | 0.882559 |
| BMI | 0.210398 | 0.096952 |
| FSIQ | 0.74649 | 0.0005 |
| PRI | 0.741272 | 0.0005 |
| PSI | 0.73018 | 0.0005 |
| VCI | 0.63494 | 0.0005 |
| WMI | 0.798105 | 0.0005 |
| Acetic acid | 0.216278 | 0.074463 |

Table S3 The content of short-chain fatty acids in fecal samples of the two groups

| **Short-chain fatty acid** | | **DS mean** | **Healthy mean** | ***p* value (<0.05)** |
| --- | --- | --- | --- | --- |
| **Acetic acid** | 969 | | 1172 | **0.03** |
| **Propionic acid** | 520 | | 456 | 0.32 |
| **Isobutyric acid** | 31 | | 48 | 0.11 |
| **Butyric acid** | 979 | | 931 | 0.48 |
| **Isovaleric acid** | 25 | | 40 | 0.092 |
| **Valeric acid** | 12 | | 9 | **0.029** |
| **Hexanoic acid** | 2.5 | | 2.5 | 0.76 |
